# Guardian Factors Affecting High Prevalence of Dental Caries in Preschool Children

**DOI:** 10.3290/j.ohpd.b2960227

**Published:** 2022-04-27

**Authors:** Haihua Zhu, Luya Lian, Kangqi Zhu, Yunxian Yu, Weifang Zhang

**Affiliations:** a Vice-Director, Stomatology Hospital, School of Stomatology, Zhejiang University School of Medicine, Clinical Research Center for Oral Diseases of Zhejiang Province, Key Laboratory of Oral Biomedical Research of Zhejiang Province, Cancer Center of Zhejiang University, Hangzhou, China. Study concept and design, consulted on statistical analysis, wrote the manuscript, read and approved the published version of the manuscript.; b Dentist, Stomatology Hospital, School of Stomatology, Zhejiang University School of Medicine, Clinical Research Center for Oral Diseases of Zhejiang Province, Key Laboratory of Oral Biomedical Research of Zhejiang Province, Cancer Center of Zhejiang University, Hangzhou, China. Study concept and design, investigation, data collection, reorganised tables and figures, revised, read and approved published version of the manuscript.; c Undergraduate Student, Zhejiang Chinese Medical University, Binjiang District, Hangzhou China. Investigation and data collection, study concept and design, read and approved published version of the manuscript.; d Professor, School of Public Health, Zhejiang University, Hangzhou, China. Data analysis and interpretation, organised tables and figures, study concept, read and approved the published version of the manuscript.; e Vice-Dean, Stomatology Hospital, School of Stomatology, Zhejiang University School of Medicine, Clinical Research Center for Oral Diseases of Zhejiang Province, Key Laboratory of Oral Biomedical Research of Zhejiang Province, Cancer Center of Zhejiang University, Hangzhou, China. Study concept, resources, supervision, project administration, read and approved the published version of the manuscript.

**Keywords:** child health, dental caries, guardians, risk factors

## Abstract

**Purpose:**

This study analysed the relationship between caregiver-related factors (dental knowledge, attitude, behaviour, and health status) and early childhood caries. It aimed to explore better intervention methods for reducing caries prevalence in preschool children.

**Materials and Methods:**

This cross-sectional investigation was carried out in Zhejiang, China. A total of 1344 guardians (parents and grandparents) paired with their children aged 3–5 years old were enrolled. The guardians completed structured questionnaires, which included their attitude, knowledge level and oral health status. The children received dental examinations. All of the data were analysed with R software. Chi-squared and Fisher’s exact tests were used to analyse different variables. Multinomial logistic regression with stepwise procedures and curve fitting was used to explore the relationship between guardians’ risk factors and the level of early childhood caries.

**Results:**

Guardians have a great influence on the incidence of early childhood dental caries. When guardians pay attention to oral health and have a good command of relevant knowledge, then the risk of early dental caries in children is low (p = 0.027). The guardians’ dental problems, e.g. dental caries (p = 0.0002), gingival bleeding problems (p = 0.049) and chewing discomfort experience (p = 0.049), demonstrated statistically significant correlations with early childhood caries levels.

**Conclusion:**

Guardians’ attitudes, knowledge, and oral health status had a statistically significant relationship with the level of early childhood caries in their children/grandchildren. Instead of instructing schoolchildren about oral health, multiple-level dental knowledge instruction of guardians is needed to prevent early childhood caries.

Early childhood caries (ECC) is considered one of the most common chronic diseases among young children. Despite significant improvement in living conditions and dental services, the prevalence and severity of caries in preschool children remains high in China. The number of decayed, missing or filled deciduous teeth (dmft) of 5-year-old children in China was 3.5 in 2005, and this number rose to 4.2 in 2015. The caries prevalence among these children increased from 66.0% in 2005 to 71.9% in 2015.^4,6,17^

Treatment of ECC often requires extensive restorative procedures, extraction of primary teeth and space maintenance. If children do not cooperate with treatment, sedation or general anesthesia may substantially increase treatment costs.^21^ Dental care is insufficient due to the lack of individual awareness and the shortage of professional dental services in China. Many families neglect regular medical care; as a result, most children go to the dental clinic only when they have dental complaints.^16^

Negligence by guardians is closely related to high ECC prevalence. For preschool children, it is the family which largely determines the environment in which they grow up. Children’s cognition and dental care behaviour are directly shaped by guardians, who play a crucial role in ECC prevalence.^10,16^ It has been proven that high caries prevalence in children was related to parents with low incomes or educational levels.^18^ Parents from less privileged backgrounds may also ignore the importance of ECC.^7^ Guardians’ supervision, e.g. making sure children brush their teeth on a daily basis, also contributes to the prevalence of ECC.^14^ Meanwhile, preschool children’s oral health status is suspected to be depended on their parents’ periodontal health status.^2^ Thus, guardians’ dental knowledge, communication with/supervision of their children, economic status, and even their own health status are decisive factors. However, few studies have focused on these risk factors from the perspective of guardians.^3^

This study presented the conceptual framework which focuses on guardians’ dental knowledge, attitudes, behaviour and oral health status. We aimed to explore the risk factors related to caries in preschool children; subsequently, precise health interventions for guardians were recommended to decrease ECC prevalence effectively.

## Materials and Methods

### Study Design

This cross-sectional investigation used a structured questionnaire and dental examination sheet: the former included guardians’ dental knowledge, attitudes, behaviour, and oral health status. The latter was mainly about the crown condition of deciduous teeth. The guardians were required to fill in the questionnaires, and their children received dental examinations from October 2018 to April 2019. This research protocol was approved by the Ethics Committee of the Chinese Stomatological Association (No. 2016–003).

Informed consent forms were submitted to the guardians. Before the survey began, guardians’ signatures were requested and checked by researchers.

Before the survey, three examiners were trained by a standard examiner in theoretical knowledge, clinical practice about inspection procedures, disease diagnosis and standard scoring. The Kappa values for each inter-examiner comparison were 0.90, 0.89 and 0.94.

A total of 1344 guardians paired with children were examined individually by three examiners and three interviewers.

### Sampling

Population data were obtained from the sixth census conducted by the National Statistics Bureau. The guardians and children were selected by multistage random cluster sampling:

1. Two districts and two counties were randomly selected from Zhejiang Province using the probability proportional to size sampling (PPS) method.
2. Three public or private kindergartens in each district or county were chosen randomly using the probability proportional to size sampling (PPS) method.
3. The 3- to 5-year-old preschool children and their guardians living in the local area for more than half a year were enrolled in the study, given that the guardians agreed to participate.

The sample size was calculated by the following formula:


$$n=\frac{t_{\alpha }^{2}\;p\;(1-p)}{d^{2}}$$

where n was the sample size, α was the level of confidence, p was the caries prevalence, set at 70.4% (according to the Fourth National Oral Health Survey of Zhejiang Province), and d was tolerance error. The non-response rate was 20%, so the minimum required sample size was 1290. A total of 1344 guardians and children were recruited in this investigation.

### Data Collection

All responses to the survey questionnaire were anonymous. Guardians were invited to take a face-to-face questionnaire verified by three trained interviewers for completeness and correctness. The questionnaires were divided into three parts: the first part reflected the guardians’ dental attitudes and knowledge; the second part asked about the guardians’ oral health status; the third part provided information about guardians’ demand to acquire dental knowledge.

All children were given dental examinations in their kindergartens. Children’s caries were diagnosed at a cavitation level by a Community Periodontal Index (CPI) probe with adequate light. The results were recorded using WHO Oral Health Survey Basic Methods (2013) criteria. Caries prevalence was assessed using the dmft index (d: decayed; m: missing/extracted; f: filled; t: deciduous teeth.

### Data Analysis

The data were analysed with R software. Guardians and children were classified into three groups according to the children’s median dmft index (MDI): dmft-free group (dmft = 0), low dmft group (0 < dmft < MDI) and high dmft group (dmft ≥ MDI). Chi-squared and Fisher’s exact tests were used to compare the variables in different groups. Curve fitting was done to show the relationship between guardians’ dental knowledge and children’s dmft index.

## Results

**Fig 1:**
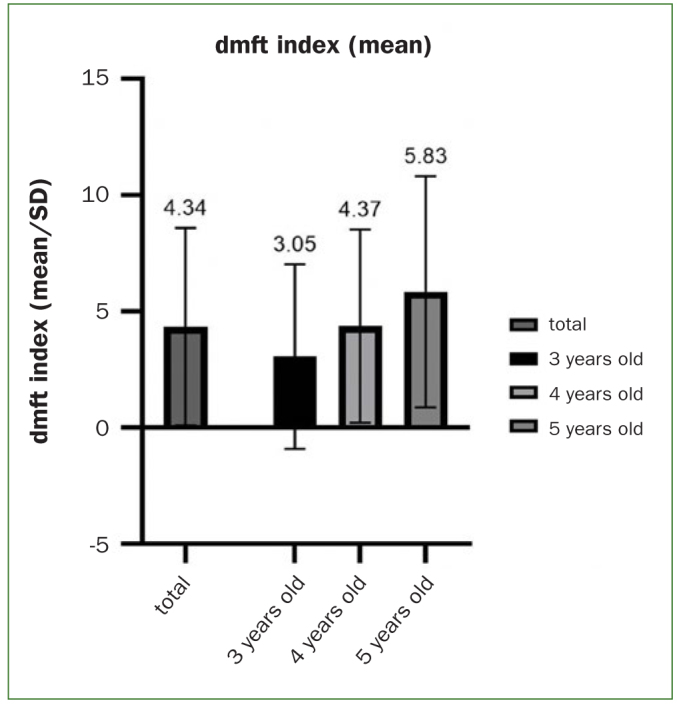
dmft index increased with age.

A total of 1344 guardians along with their children were enrolled. The mean dmft index of children (mean ± SD) was 4.34 ± 4.25; the median dmft index (MDI) was 4.73; the caries prevalence (dmft > 0) was 72.1%. The dmft index increased with age. The mean dmft index of each group is shown in Fig 1.

**Fig 2:**
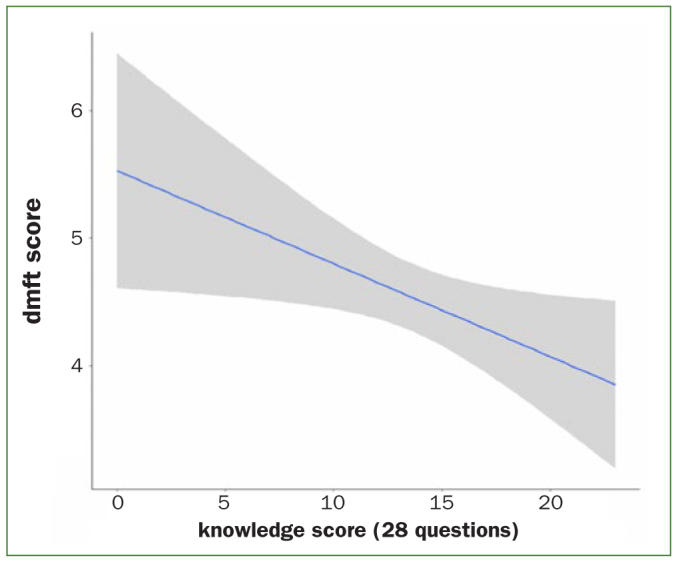
Relationship between guardian factors and ECC.

We quantified the guardians’ attitudes and knowledge and then related the score with the children’s dmft index. The curve-fitting result indicates that guardians with higher awareness and better knowledge of children’s oral health could reduce the ECC risk (p = 0.027) (Fig 2). Guardians’ awareness of cleaning their children’s gums with gauze before teeth erupt (p = 0.0001), using clean gauze to wipe newly erupted teeth routinely, and giving children warm water to gargle or drink after meals (p = 0.005) reduced children’s risk of ECC. Furthermore, guardians’ awareness of providing beverages in a cup instead of using baby bottles after the age of 1-year would decrease the risk of getting ECC (p = 0.044) (Table 1).

**Table 1:** The relationship of guardians’ attitudes and knowledge and ECC (N = 1344)

Variable	Level	Total	dmft, n (%)			p-value
			0	1–4	≥5	
1. Oral health is important to the quality of life.						
	Agree	1300	371 (98.93)	433 (96.22)	496 (95.57)	0.051*
	Disagree	9	1 (0.27)	3 (0.67)	5 (0.96)	
	Uncertain	35	3 (0.80)	14 (3.11)	18 (3.47)	
11. Fluoride is useless for protecting teeth.						
	Agree	84	21 (5.60)	30 (6.67)	33 (6.36)	0.226
	Disagree	383	119 (31.73)	134 (29.78)	130 (25.05)	
	Uncertain	877	235 (62.67)	286 (63.56)	356 (68.59)	
18. Gums should be cleaned with clean gauze before teeth erupt.						
	Do not know	723	180 (48.00)	223 (49.56)	320 (61.66)	0.0001
	Know	621	195 (52.00)	227 (50.44)	199 (38.34)	
20. Newly erupted teeth should be cleaned by clean gauze routinely, and children should be given warm water to gargle or drink after meals.						
	Do not know	622	159 (42.40)	194 (43.11)	269 (51.83)	0.005
	Know	722	216 (57.60)	256 (56.89)	250 (48.17)	
22. Children should drink beverages in a cup, instead of using baby bottles after the age of 1 year.						
	Do not know	570	144 (38.40)	181 (40.22)	245 (47.21)	0.044
	Know	774	231 (61.60)	269 (59.78)	274 (52.79)	
25.Toothpastes with fluoride can prevent caries.						
	Do not know	880	247 (65.87)	280 (62.22)	353 (68.02)	0.164
	Know	464	128 (34.13)	170 (37.78)	166 (31.98)	
27. Fluoride and calcium supplements are very important to oral health. Infants and young children should be provided with fluoride and calcium under the guidance of doctors.						
	Do not know	875	244 (65.07)	280 (62.22)	350 (67.44)	0.072
	Know	470	131 (35.93)	170 (37.78)	169 (32.56)	

*Fisher’s exact test; others are chi-squared test.

Regarding questions about their own oral health, guardians who had caries (p = 0.0002), gingival bleeding (p = 0.049), and chewing discomfort (p = 0.027) were correlated with a high dmft index in their children (Table 2).

**Table 2:** The relationship between guardians’ own oral health status and ECC (N = 1340)

Variable	Level	dmft, n (%)			p-value
		0	1–4	≥5	
**Self-assessment of oral health status** 1. Guardians self-rating of their oral health					
	Poor	37 (9.89)	63 (14.03)	83 (16.05)	0.063
	Moderate	205 (54.81)	238 (53.01)	283 (54.74)	
	Good	132 (35.29)	148 (32.96)	151 (29.21)	
2. Guardians satisfied with their measures to maintain oral health					
	Poor	45 (12.03)	59 (13.14)	75 (14.51)	0.593
	Moderate	198 (52.80)	242 (53.90)	290 (56.09)	
	Good	131 (35.03)	148 (32.96)	152 (29.40)	
**Oral health status** 3. Do the guardians have dental caries?					
	Uncertain	32 (8.56)	38 (8.46)	60 (11.61)	0.0002
	No	193 (51.60)	217 (48.33)	200 (38.68)	
	Yes	149 (39.84)	194 (43.21)	257 (49.71)	
4. Do the guardians have gingival bleeding?					
	Uncertain	19 (5.08)	24 (5.35)	42 (8.12)	0.049
	No	147 (39.30)	182 (40.53)	206 (39.85)	
	Yes	208 (55.61)	243 (54.12)	269 (52.03)	
5. Do the guardians suffer from toothaches?					
	Often	20 (5.35)	31 (6.90)	24 (4.64)	0.272
	Sometimes	130 (34.76)	172 (38.31)	179 (34.62)	
	Rarely	118 (31.55)	139 (30.96)	186 (35.98)	
	Never	106 (28.34)	107 (23.83)	128 (24.76)	
6. Do the guardians suffer from chewing discomfort?					
	Often	16 (4.28)	36 (8.02)	36 (6.96)	0.027
	Sometimes	115 (30.75)	136 (30.29)	149 (28.82)	
	Rarely	96 (25.67)	137 (30.51)	172 (33.27)	
	Never	147 (39.30)	140 (31.18)	160 (30.95)	
7. Do the guardians’ oral symptoms affect their quality of life?					
	Often	20 (5.35)	32 (7.13)	32 (6.19)	0.243
	Sometimes	89 (23.80)	119 (26.50)	131 (25.34)	
	Rarely	100 (26.74)	145 (32.29)	162 (31.33)	
	Never	165 (44.12)	153 (34.08)	192 (37.14)	
**Oral health behavior** 8. Toothbrushing frequency of the guardians					
	Never	6 (1.60)	4 (0.89)	8 (1.55)	0.655
	Once a month	3 (0.80)	3 (0.67)	7 (1.35)	
	Once a week	5 (1.34)	14 (3.12)	9 (1.74)	
	Once a day	230 (61.50)	281 (62.58)	318 (61.51)	
	Several times a day	130 (34.76)	147 (32.74)	175 (33.85)	
9. Flossing frequency of the guardians					
	Never	330 (88.24)	397 (88.42)	452 (87.43)	0.464
	Once a month	10 (2.67)	16 (3.56)	16 (3.09)	
	Once a week	17 (4.55)	15 (3.34)	27 (5.22)	
	Once a day	9 (2.41)	18 (4.01)	16 (3.09)	
	Several times a day	8 (2.14)	3 (0.67)	6 (1.16)	
10. Mouthrinsing frequency of the guardians					
	Never	244 (65.24)	287 (63.92)	331 (64.02)	0.580
	Once a month	21 (5.61)	14 (3.12)	24 (4.64)	
	Once a week	18 (4.81)	21 (4.68)	31 (6.00)	
	Once a day	70 (18.72)	95 (21.16)	93 (17.99)	
	Several times a day	21 (5.61)	32 (7.13)	38 (7.35)	

Chi-squared test.

**Fig 3:**
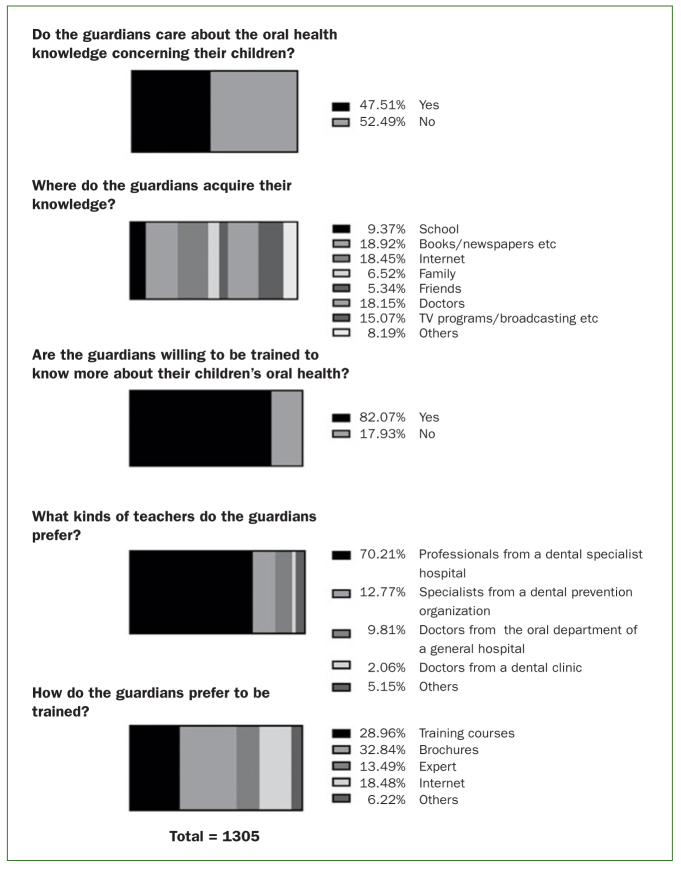
Guardians’ demand for acquiring dental knowledge.

In terms of guardians’ demands for oral health education, 82.07% of the guardians were eager to receive the relevant dental instructions. Among those who received some education on dental knowledge, they acquired their knowledge from books/newspapers (24.4%), TV programmes (19.5%), internet (23.8%), dentists (23.5%) and schools (12.1%). Concerning the children’s oral health education demands, 36.0% of the guardians were willing to attend a training course, 40.8% preferred reading brochures, and 23.0% were interested in surfing the internet for the necessary information; the expert hotline was selected by 16.8% of the guardians. Considering the type of trainers, 78.4% of the guardians put trust in professionals from a dental specialist hospital, 14.3% preferred being instructed by specialists from a dental prevention organisation, and 11.0% were willing to be instructed by doctors from a dental department of a general hospital. Only 2.3% of them chose doctors from a private dental clinic, as shown in Table 3 and Fig 3.

**Table 3:** Guardians’ demand for acquiring dental knowledge (N = 1305)

Variable	N (%)
1. Do the guardians care about oral health knowledge concerning their children?	
Yes	620 (47.51%)
No	685 (52.49%)
2.Where do the guardians get their knowledge?	
School	158 (12.11%)
Books/newspapers etc	319 (24.44%)
Internet	311 (23.83%)
Family	110 (8.43%)
Friends	90 (6.90%)
Doctors	306 (23.45%)
TV programs/broadcasting etc	254 (19.46%)
Others	138 (10.57%)
3. Are the guardians willing to be trained to know more about their children’s oral health?	
Yes	1071 (82.07%)
No	234 (17.93%)
4. What kinds of teachers do the guardians prefer?	
Professionals from a dental specialist hospital	1023 (78.39%)
Specialists from a dental prevention organisation	186 (14.25%)
Doctors from the oral department of a general hospital	143 (10.96%)
Doctors from a dental clinic	30 (2.30%)
Others	75 (5.75%)
5. How do the guardians prefer to be trained?	
Training courses	470 (36.02%)
Brochures	533 (40.84%)
Expert hotline	219 (16.78%)
Internet	300 (22.99%)
Others	101 (7.74%)

## Discussion

This study presents a comprehensive frame for investigating the relationship between guardian-related factors and ECC. The caries prevalence of children aged 3–5 was high (72.1%) and was statistically significantly correlated with increasing age, which might be due to the continuous and cumulative ECC. The study exhibited a strong relationship between guardian-related factors and ECC. Guardians’ attitudes and knowledge levels are directly related to the severity of early childhood caries. The oral health status of guardians is consistent with that of their children. It might be more effective to educate guardians rather than their children.

Previous research has already proven the relationship between parental attitudes/knowledge and children’s caries prevalence.^8,13^ Parental education improves children’s knowledge and promotes oral hygiene practices. Parents’ decisions have far-reaching consequences for their children’s whole life.^1,15^ Substantial knowledge differences exist between different guardians, and it is recommended that they turn to professional institutions or specialists for help.^20^

Guardians decide how/what children eat and may also pass on their oral hygiene routines to the next generation. Parents’ cariogenic microorganisms may be transmitted to their children in various ways. It was shown that children tend to have a dmft index that corresponds to mothers’ caries status.^11,19^

Our study further indicated the strong relationship between guardians and their children’s ECC: less knowledge, negative attitude, and poor oral health of guardians may increase the likelihood of developing ECC.

In terms of guardians’ oral health, our study found that the guardians of children with a high ECC prevalence usually have poor oral health, mainly as follows: dental caries, gingival bleeding, or chewing discomfort. Untreated oral health problems reflected guardians’ ignorance of oral problems and neglect of oral cleaning, which might affect their children’s oral health. Guardians’ oral health status depends on their behaviour and attitudes, which was proven to influence the risk of ECC of the next generation. There are many links between guardians’ poor oral health and children’s ECC; the relationship between them is illustrated in Fig 2.

Interestingly, despite fluoride’s critical role in preventing caries of children aged 1-3, this study found that guardians’ knowledge about oral hygiene, e.g. fluoride supplementation, was not statistically significantly correlated with ECC prevalence. At the same time, the application of fluoride varnish has been established as a cost-effective community strategy to prevent ECC.^9,22,23^ This implies that guardians lacked correct knowledge and sufficient education in the dental field. According to Petersen, China is increasingly emphasising oral health education for school children.^12^ However, in most cases, it may be that only after children suffer from ECC do the guardians begin to acquire oral health-related knowledge. It is perhaps not easy to make children aware of the importance of protecting teeth, which prompts us to find new approaches to preventing ECC.

Preschool children have limited self-care ability, so guardians (as the closest persons) should instruct them. Parents and other family members should actively participate in ECC prevention or treatment.^5^ It is suggested that guardians be trained in relevant knowledge instead of directly educating preschool children.

Although lack of related dental knowledge is common, our study shows that most guardians in Zhejiang Province were eager to receive training (82.1%). How to conduct proper training in a convenient way should be emphasised in the future. Concerning the training method, guardians’ preference varied considerably, but reading brochures was the most common preference. Popularising comprehensive dental knowledge in health brochures for newborns and distributing these brochures among guardians is necessary. We must further explore other effective methods of publicity.

The limitations of this study included recall bias and comprehension bias of the interviewees, which might have resulted in a few false-negative results in the survey. Multi-level questionnaires should be designed for future studies and be confirmed at several levels to reduce recall bias.

## Conclusion

The study showed a high ECC prevalence in children aged 3–5 years old in Zhejiang Province, China. Guardians’ poor dental knowledge, negative attitude, poor supervision, bad habits, and poor oral health status may lead to ECC. Besides properly educating preschool children, it is even more urgent to instruct guardians on the details of dental health.
